# Biological Functions of Type II Toxin-Antitoxin Systems in Bacteria

**DOI:** 10.3390/microorganisms9061276

**Published:** 2021-06-11

**Authors:** Muhammad Kamruzzaman, Alma Y. Wu, Jonathan R. Iredell

**Affiliations:** 1 Centre for Infectious Diseases and Microbiology, The Westmead Institute for Medical Research, The University of Sydney, Westmead, NSW 2145, Australia; alwu9896@uni.sydney.edu.au; 2 Westmead Hospital, Westmead, NSW 2145, Australia

**Keywords:** toxin-antitoxin system, mobile genetic elements, stress response, plasmid maintenance, bacteria virulence, biofilm

## Abstract

After the first discovery in the 1980s in F-plasmids as a plasmid maintenance system, a myriad of toxin-antitoxin (TA) systems has been identified in bacterial chromosomes and mobile genetic elements (MGEs), including plasmids and bacteriophages. TA systems are small genetic modules that encode a toxin and its antidote and can be divided into seven types based on the nature of the antitoxin molecules and their mechanism of action to neutralise toxins. Among them, type II TA systems are widely distributed in chromosomes and plasmids and the best studied so far. Maintaining genetic material may be the major function of type II TA systems associated with MGEs, but the chromosomal TA systems contribute largely to functions associated with bacterial physiology, including the management of different stresses, virulence and pathogenesis. Due to growing interest in TA research, extensive work has been conducted in recent decades to better understand the physiological roles of these chromosomally encoded modules. However, there are still controversies about some of the functions associated with different TA systems. This review will discuss the most current findings and the bona fide functions of bacterial type II TA systems.

## 1. Introduction

Toxin-antitoxin systems are small genetic elements made up of two genes; one producing a toxin and another producing its antidote to neutralise the cognate toxin in the bacterial cell. The toxin is very stable, while the antitoxin is labile and degraded in the plasmid-free cells. The stable toxin then exerts its toxic effects to kill or inhibit the growth of the plasmid-free cell [1]. In the case of chromosomal TA systems, different stresses induce the expression of bacterial proteases that efficiently degrade labile antitoxin. Unopposed toxins can kill or inhibit cell growth by targeting a variety of important cellular processes, including DNA replication, translation, membrane integrity, cytoskeleton formation and cell-wall synthesis [2,3,4].

The first toxin-antitoxin (TA) system, *ccdA-ccdB*, was discovered on an IncF plasmid in 1983 as a plasmid maintenance system, acting through post-segregational killing of plasmid-free cells [5]. After that, more plasmid-encoded TA systems were identified by their ability to enhance plasmid stability. At the same time, thousands of TA systems were identified on bacterial chromosomes. Different bioinformatic approaches [6,7] allowed the identification of numerous TA systems, and today, more than 10,000 (putative) TA modules are known. The role of plasmid-mediated TA systems is easy to predict, i.e., the maintenance of plasmids, but the functions of chromosomal TA systems were mysterious for a long time and thought to be selfish genetic elements. To date, experimental evidence suggests that both chromosomal and plasmid-mediated TA systems are involved in different physiological functions of bacteria [2,3,4,8,9,10,11,12,13,14]. The high abundance of type II TAS in bacteria and their proven involvement in the regulation of bacterial physiological conditions, including survival in adverse environments, virulence, biofilm formation, resistance to bacteriophages etc., have increased interest in TA system research considerably. The precise biological functions of many of the type II TA systems have been elucidated, but there is still confusion and controversy surrounding some of the identified functions of type II TA systems, including their roles in antibiotic tolerance and persistence. Many more biological functions associated with type II TA systems have been experimentally confirmed, with the discovery of new TA systems and their associated functions making the area of TA research more interesting and attractive to the scientific community. Here, we bring together all recent updates about the roles of type II TAS and discuss the biological functions encoded by this important TA type.

## 2. TA Systems Biology and Classification

**Figure 1:**
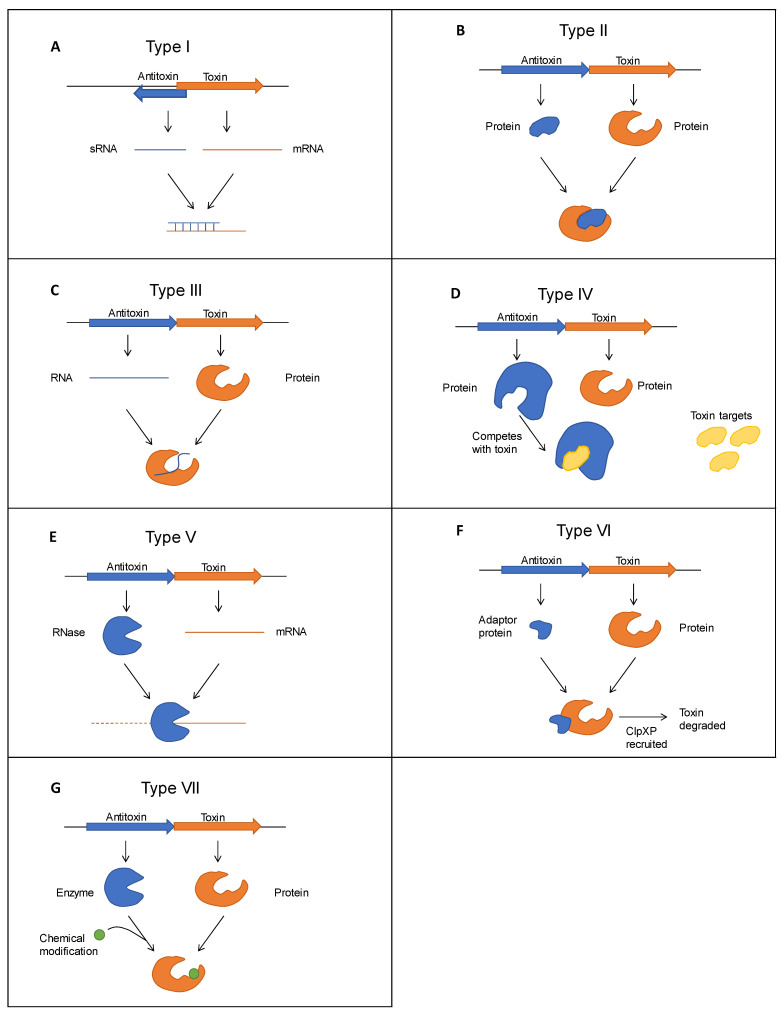
Major types of toxin-antitoxin systems (**A**–**G**). Toxins are shown in orange and antitoxins in blue. Type I (**A**): the antitoxin antisense RNA base pairs with toxin mRNA and inhibits translation. Type II (**B**): antitoxin protein binds with toxin protein and inhibits its activity. Type III (**C**): the antitoxin sRNA directly binds to the toxin protein and inhibits its activity. Type IV (**D**): antitoxin protein binds to the toxin target and protects from toxic effects. Type V (**E**): RNase antitoxin specifically degrades toxin mRNA. Type VI (**F**): antitoxin adaptor protein binds to the toxin and promotes its degradation by cell proteases. Type VII (**G**): the enzymatic function of antitoxin modifies the toxin protein to a non-toxic one.

To understand the biological role of type II TA systems, it is imperative to understand the basic mechanism of action of type II TA systems and how this differs from other TA types. Currently, TA systems are classified into seven major types [10,15] based on the nature of the antitoxin molecules and their mechanism of action in neutralising the toxin (Figure 1). For all types of TA systems described, the toxins are proteins, while the antitoxins are either proteins or, in the case of type I and type III TA systems, non-coding RNAs. For type I, the antisense RNA of the antitoxin directly binds to the toxin mRNA and block the translation of toxin, but in type III TA systems, the antitoxin RNA directly interacts with the toxin protein to neutralise its function. For all other TA systems, the antitoxins are proteins, but their mechanisms of action differ. In type II TA systems, a direct protein-protein interaction between toxin and antitoxin forms a TA complex that blocks the action of the toxin. In contrast, the antitoxins of the type IV TA system do not need direct interaction, but both toxin and antitoxin compete for a common target binding site. Two single instances of TA modules with mechanisms of action different from type I–IV TA systems have been described as type V and type VI TA systems, respectively [10,16]. The type V TA system, GhoT/GhoS, is distinguished by the enzymatic activity of the antitoxin GhoS, which specifically cleaves the toxin mRNA and blocks its translation [17], while the type VI antitoxin SocA acts as an adaptor protein to direct the toxin SocB to proteolysis, leading to SocB inactivation [18]. Type VII TA systems have also been recently described, and at least three different TA modules (*Hha/TomB*, *TglT/TakA* or (*MenT3/MenA3*), *HepT/MntA*) have been categorised as type VII TA system [15]. In this type, the antitoxin is an enzyme that modifies the toxin to neutralise its activity.

## 3. Biological Functions of Type II TA Systems

**Figure 2:**
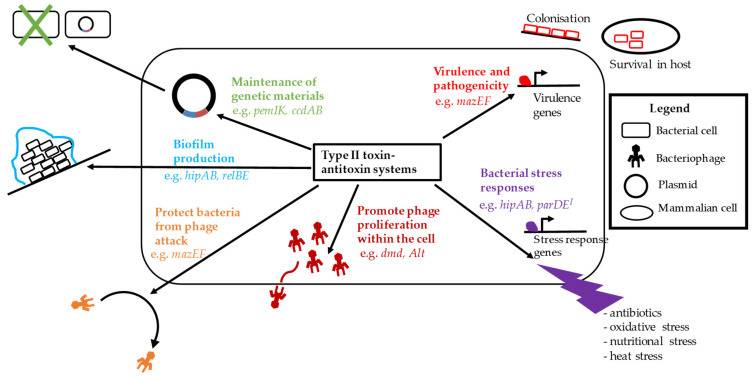
Summary of biological functions of bacterial type II TA systems.

Type II TA systems are the best studied of all the seven types and are widely distributed in bacterial chromosomes as well as mobile genetic elements like plasmids and pathogenicity islands (PAIs). Toxins of type II TA systems include RNases, kinases, and acetyltransferases and play important roles in the maintenance of genetic materials, bacterial virulence, biofilm formation, phage inhibition and different types of stress management, including antibiotic tolerance and persister formation. These functions are summarised in Figure 2. We will discuss here all these biological functions associated with type II TA systems in bacteria.

### 3.1. Maintenance of Genetic Materials

#### 3.1.1. Plasmid Maintenance

Perhaps the best-known of the various functions of plasmid-borne type II TA systems is in plasmid maintenance; indeed, TA systems are sometimes also known as “plasmid addiction systems” or “plasmid maintenance systems”. The two earliest discovered TA systems, *ccdAB* [19,20] and *hok-sok* [21] were both found to mediate this through post-segregational killing (PSK) of plasmid-free cells and in subsequent years many other TA systems with the same function have been discovered [22,23,24,25,26]. Recently this idea of plasmid maintenance through PSK has been questioned, with some arguing that there is insufficient evidence for cell death [27] and that toxin concentrations in experimental settings are higher than the physiological concentration [19,22]. Rather, the plasmid maintenance function may be the result of growth inhibition [27]. Further experimental work with physiologically relevant concentrations of toxins is needed.

**Figure 3:**
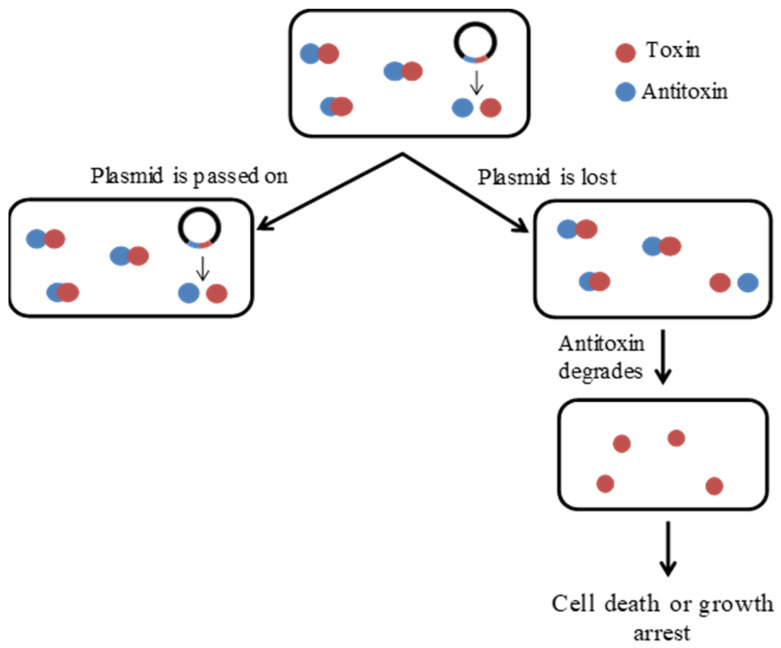
Plasmid maintenance mechanism of type II TA systems through post-segregational killing or bacterial growth arrest.

Regardless of whether TA system toxins lead to cell death or growth inhibition (and indeed, given the variety of TA systems that have been discovered, both may be true), the mechanism by which plasmid maintenance by plasmid-borne TA systems occurs generally follows the same principle. A typical TA system consists of a stable toxin and a more unstable antitoxin. During normal growth, the classical antitoxin forms a complex with the toxin to block its effects (on PSK or growth inhibition). Following cell division, the TA complex is passed on to daughter cells; if the daughter cell does not inherit a copy of the plasmid, it is unable to produce any more toxin or antitoxin molecules [1,24,28]. It has long been held that the unstable antitoxins are then preferentially degraded, for example, by proteases such as the Lon protease in the case of type II TA systems, freeing the toxins to exert their cytotoxic or growth inhibitory effects (Figure 3) [1,10,28,29,30].

However, recently it has been argued that toxin activation is more likely the result of preferential degradation of the antitoxin mRNA left in the cell, followed by de novo synthesis of new toxin proteins from the remaining toxin mRNA [31]. This is indeed the case with the *mqsRA* and *ghoST* systems, with the toxin MqsR preferentially cleaving the antitoxin GhoS transcript [32], but this system is unusual as it involves one TA system regulating another TA system, and the *ghoST* system has also been classified as a type V TA system [17]. Data on the relative stabilities of toxin and antitoxin transcripts of more conventional TA systems are yet unavailable and, along with studies investigating the degradation rates of bound vs. unbound antitoxin proteins, are needed before the true processes leading to the observed excess of toxin protein compared to antitoxin can be confirmed.

Not all plasmid-borne TA systems are equal in their plasmid maintenance ability, with some being specialised to certain strains or species. For example, although *pemIK* is able to mediate plasmid maintenance in multiple strains of both *Escherichia coli* and *Klebsiella pneumoniae*, the ability of *ccdAB* to do the same varies with the host strain [33]. These differences appear to be the result of sequence variations [33], with a single amino acid substitution being sufficient to render the CcdB toxin inactive [34]. It is possible that these alternative versions are involved in other processes within the cell, similar to their chromosomally located cousins [2,35,36].

#### 3.1.2. Maintenance of Other Genetic Elements

The ability of TA systems to stably maintain genetic elements is not limited to plasmids. Multiple TA systems have also been implicated in the maintenance of genomic islands, transposons, integrative conjugative elements (ICEs) and secondary chromosomes, though research in this area is still scarce compared to plasmids.

Genomic islands are sections of the bacterial genome that show evidence of horizontal transfer. They often differ significantly even between two closely related strains, and if not currently mobile, show evidence of past mobility. Importantly, genomic islands also often carry other genes that can offer selective advantages to their hosts and can thus be described by the functions of these genes (e.g., pathogenicity islands carry virulence genes, resistance islands carry antibiotic resistance genes etc.) [37]. Maintenance of genetic islands by TA systems has been reported. For example, the type II TA system *sezAT* is found within the SsPI-1 pathogenicity island in *Streptococcus suis*, stabilising the island and preventing the elimination of excised SsPI-1 from the cell [38]. The TA system *sgiAT* also prevents the loss of the resistance island SGI1 in *Salmonella* (among other species), maintaining it within the cell in the presence of IncA/C plasmids, which would otherwise trigger excision of SGI1 and its subsequent loss due to incompatibility between the plasmid and SGI1 [39]. A novel TA system *mosAT* in *Vibrio cholerae* was shown to be responsible for the stable maintenance of the SXT integrative conjugative element [40]. The SXT is a ~100 kb ICE that confers resistance to multiple antibiotics in clinical isolates of this organism. The *mosAT* system has low transcriptional levels when SXT is integrated; however, its expression is increased when SXT is in an extrachromosomal state and vulnerable to loss [40].

TA systems are also present on chromosome II of *Vibrio cholerae*. This secondary chromosome is smaller than chromosome I, but it encodes some essential genes and is vital for *Vibrio cholerae* survival. All identified TA systems (at least 17) on this chromosome are found within the same sedentary chromosomal integron (SCI), also referred to as superintegron (SI), including 3 homologues of *parDE* that have been found to contribute to the stability of the chromosome in a manner similar to that of plasmid-mediated post-segregational killing [41]. It was demonstrated that two type II TA systems, *relBE1* and *parDE1*, located in the SCI of *Vibrio vulnificus,* were responsible for the stability of this SCI [42].

Various TA systems in other types of genomic islands have also been identified, though the functionality of these systems has yet to be confirmed. These include several TA systems identified within a genomic island in *Vibrio diabolicus* [43], as well as putative TA systems of various types in close proximity to genomic islands in adherent-invasive *E. coli* (AIEC) [44]. Similarly, at least six different TA systems have been found to be associated with *Tn3* family transposons. Although their exact function within the transposon has yet to be confirmed experimentally, it has been speculated that they play a vital role in maintaining the transposons, given the proximity of the TA system genes to the core transposase and resolvase genes, rather than further out like most *Tn3* passenger genes [45]. An analysis of chromosomally encoded TA systems in *Acidithiobacillus ferrooxidans* also found that many were encoded within or near mobile genetic elements. Two of these, found within ICE*Afe*1, were found to be functional when expressed in trans in *E. coli*; and it was thought that these TA systems are involved in the maintenance of these mobile genetic elements [46].

Type II TA systems are also found to be responsible for the stability of prophages. A type II TA system *ParESO/CopASO* in the cryptic prophage CP4So in *Shewanella oneidensis*, stabilises the circular prophage in the host bacteria after its excision [47]. Furthermore, some prophages also have toxin-antitoxin (TA) systems for stabilising and self-preservation of the phage [26].

### 3.2. Type II TAS in Bacterial Virulence and Pathogenesis

Type II TA systems are largely associated with increased bacterial colonisation in host organs. Evidence suggests that there is a direct association between the presence of an increased number of TA systems in a bacterial strain and an increase in virulence and pathogenesis caused by that bacterial strain. For example, pathogenic *Mycobacterium tuberculosis* has about 90 putative TA systems [48], while the non-pathogenic *Mycobacterium smegmatis* has only two putative TA systems [13]. Pathogenic *Salmonella* Typhimurium has at least 11 TA systems, but these are absent from other non-pathogenic strains [49]. The chromosomal and plasmid-borne type II TA systems involved in bacterial virulence are summarised in Table 1.

#### 3.2.1. Virulence and Pathogenesis Mediated by Plasmid-Borne Type II TA Systems

Many virulence plasmids of pathogenic bacteria have type II TA systems, and these contribute to the virulence and pathogenesis of different bacterial species either by the maintenance of virulence plasmids in pathogenic bacteria or by directly participating in pathogenesis (Table 1). A large ~210-kb plasmid, pINV, is crucial for virulence in all four *Shigella* species [76]. pINV carries a pathogenicity island that encodes a type three secretion system (T3SS) and the virulence gene *icsA,* which is involved in adhesion and actin-mediated motility [77,78]. pINV also carries three type II TA systems, *mvpAT/vapBC*, *ccdAB* and *gmvAT*, which are directly involved in the maintenance of the virulence plasmid in different conditions. *mvpAT* is a member of the *vapBC* family and contributes to plasmid maintenance at temperatures found in the environment and the human intestine [50,51], while *gmvAT* contributes significantly to pINV stability at 21 °C [50]. The *Salmonella* virulence plasmid pSLT (50–90-kb) possesses two type II TA systems, *vapBC* and *ccdAB*, and both are found to contribute to the stability of this plasmid [52]. In addition, *vapBC_ST_* promotes the survival of *Salmonella* bacteria inside infected host cells [9,52,53]. It was also found that plasmid-mediated TA systems have a prominent role in preserving plasmid integrity and ensuring the maintenance of virulence genes in free-living conditions of *Pseudomonas syringae* [79]. Another plasmid-mediated TA system, *pumAB,* found in the *Pseudomonas aeruginosa* plasmid pUM505, contributes to the virulence of this bacteria. *P. aeruginosa* strains with elevated expression of PumA toxin improved mouse organ invasion and increased *Caenorhabditis elegans* and mouse mortality rates [54,55]. The *pemIK*_Sa_ TA system from the *S. aureus* plasmid pCH91 participates in the global regulation of *Staphylococcal* virulence by altering the translation of large pools of genes [56]. Plasmid-mediated type II TA systems thus appear to contribute to bacterial virulence in several different ways. Not only do they maintain virulence plasmids, but these plasmid-mediated type II TA systems also promote survival advantages to the bacteria in different physiological and environmental conditions and may be important in controlling the expression of virulence and virulence-associated genes. Plasmid-mediated TA systems are enriched in *E. coli* bacteria carrying the extended-spectrum β-lactamase (ESBL) gene that confers resistance to major β-lactam antibiotics [80,81,82]. Among the ESBL gene types, *E. coli* carrying the most prevalent *bla*_CTX-M-15_ gene has more TA systems than those carrying other ESBL gene types [81,82]. The type II TA system *vagCD* was exclusively associated with the most successful pathogenic *E. coli* clone ST131, which has a *bla*_CTX-M-15_ carrying plasmid [81], and IncF plasmids in *E. coli* have more TA systems than other Inc type plasmids [80]. This suggests the possible link between the TA systems, antibiotic resistance genes, plasmid types and pathogenic bacterial clones.

#### 3.2.2. Virulence and Pathogenesis Mediated by Chromosomal Type II TA Systems

Type II TA systems are abundant in bacterial chromosomes and have been shown to participate in pathogenesis either by increasing the survival of the pathogen inside the host or by inducing the virulence factors (Table 1). *M. tuberculosis* is a deadly human pathogen, and the genome of this bacteria carries 90 TA systems. The majority of these (50/90) are members of the virulence-associated protein (*vapBC*) type II TA family [61]. Among them, several *vapBC* TA systems are involved in the infection and pathogenesis of this bacteria. Deletion of *vapBC3* and *vapBC4* impairs the ability of *M. tuberculosis* to infect hosts in animal models [60]. *vapBC11* was found to be upregulated during exposure to different antibiotics [60,83] and was also essential for *M. tuberculosis* to establish infection in guinea pigs [61]. The *M. tuberculosis* genome carries at least nine *mazEF* family type II TA systems, and among them, *mazEF3*, *mazEF6* and *mazEF9* contribute to *M. tuberculosis* virulence [62]. Inactivation of three of these *M. tuberculosis* *mazEF* TA systems reduced persistence in vitro, survival in macrophages, and cell numbers in the spleen and lungs of guinea pigs [62]. The *mazEF* TA system in *S. aureus* promotes biofilm antibiotic tolerance and plays a crucial role in transitioning from acute to chronic *S. aureus* infection [63]. The loss of *S. aureus mazF* resulted in an increased bacterial burden and decreased survival rate of mice compared to the wild-type bacteria [63].

Chromosomal type II TA systems also play an important role in the persistence of *Salmonella* Typhimurium in macrophages in a mouse model for typhoid fever [84] and inside fibroblasts and epithelial cells [53]. One antitoxin, *sehB* of the *sehAB* type II TA system in *Salmonella enterica,* can induce the virulence of this organism in mice [85]. It was found that eight single amino acid mutations (Y32, L42, L52, I60, S107, L121, L129, and F140) in SehB attenuates the virulence of *S. enterica* in the mouse model. These amino acids are important for SehB auto-repression, homodimerisation and DNA binding activity, all of which were found to be required for the virulence of *S. enterica* [85].

Three type II TA systems were also found to contribute to the virulence of non-typeable *Haemophilus influenzae* (NTHi) [64,65], which is a Gram-negative organism and the second most common cause of acute otitis media after *Streptococcus pneumoniae*. Inactivation of the *vapBC-1* and *vapXD* TA systems reduced the survival of NTHi inside epithelial cells and in the ear of infected chinchillas [64]. The toxin VapC of this *vapBC* TA system is a PIN domain-containing protein, and it was recently identified that mutations in the conserved amino acids of the PIN domain of VapC1 of NTHi are associated with decreased toxicity in *E. coli* and decreased NTHi survival during infections of primary human tissues ex vivo [86]. Another TA system, the *higBA*-like module *toxAvapA* of NTHi, also contributes to NTHi survival following infection of epithelial cells of the upper respiratory tract and plays an important role during chinchilla middle ear infection [65].

The genome of uropathogenic *E. coli* (UPEC) has fewer TA systems than non-pathogenic *E. coli*, an exception to the rule that pathogenic bacteria possess more TA systems. Three type II TA systems in UPEC are involved in the successful colonisation of the bacteria in the host environment. Two of them, *ybaJ-hha* and *yefM-yoeB*, contribute to colonisation in the bladder, while another TA system, *pasTI*, contributes to colonisation in mouse kidneys [66].

*Vibrio cholerae* is the causative agent of the devastating diarrhoeal disease, Cholera. *V. cholerae* has two chromosomes, with the smaller chromosome (chromosome II) carrying seven *relBE* TA systems. Deletion of *relBE4* and *relBE7* reduced colonisation in the mouse intestine, suggesting roles in intestinal colonisation and virulence [67]. The chromosomal *fitBA* type II TA system in *Neisseria gonorrhoeae* (a sexually transmitted human pathogen) may act as an intracellular growth regulator of this pathogen. Disruption of *fitBA* enhanced the intracellular proliferation of the bacteria, this being associated with increased their ability to cross the epithelial cell layer [68].

The ε–ζ TA system is widely distributed in the plasmids and chromosomes of both Gram-positive and Gram-negative bacteria [58]. The plasmid-encoded ε–ζ contributes to plasmid maintenance by killing plasmid-free cells, but its chromosomal homologue *pezAT*, located in the *pneumococcal* pathogenic island PPI-1 [57,87] was shown to be required for the establishment of full virulence in mice [58,69]. The toxin ζ/PezT specifically phosphorylates the peptidoglycan precursor UDP-N-acetylglucosamine (UNAG) at the 3′-OH group of the N-acetylglucosamine moiety and produces UNAG-3P, which inhibits peptidoglycan synthesis. At different stress conditions, the toxin PezT is released from the antitoxin PezA, which then enhances the production of UNAG-3P. Increased UNAG-3P production results in inhibition of cell wall synthesis and lysis of the *pneumococcus* populations [58]. Lysis of Gram-positive bacteria accelerates the release of cellular components such as virulence factors, teichoic acids, lipoteichoic acids, and bacterial DNA [88], which are detrimental to the infected host. Cell fragments from lysed *pneumococcus* inhibit phagocytosis and impair phagocyte-mediated defence against living *pneumococcus* [69]. Most importantly, *pneumococcus* lysis leads to the triggered release of pneumolysin (Ply), the major virulence factor of this organism [89].

#### 3.2.3. Type II TA Systems That Negatively Regulate Virulence

Type II TA systems are also involved in the attenuation of the virulence of bacteria. The chromosomal *higBA* TA system in *P. aeruginosa* is involved in the reduction of virulence in this species. Activation of the toxin HigB reduces the production of the *P. aeruginosa* virulence factors pyochelin and pyocyanin and reduces swarming motility [59]. Swarming motility is involved in biofilm formation, bacterial virulence and pathogenesis. Pyochelin is a siderophore produced by *P. aeruginosa,* which increases the growth and lethality of pathogenic bacteria [90], and several studies have linked pyochelin and virulence [90,91,92]. Pyocyanin is a blue redox-active secondary metabolite that interferes with multiple cellular functions and plays crucial roles in *P. aeruginosa* infections [93]. Another type II TA system, *savRS* in the *S. aureus* chromosome, negatively regulates the virulence and pathogenicity of this bacteria. Genetic deletion of the *savRS* system led to increased hemolytic activity and pathogenicity in a mouse subcutaneous abscess model. Furthermore, it was found that SavR and SavRS can directly bind to the promoter region of two virulence genes *hla* and *efb* and repress their expression [70].

In most cases, the overexpression of the TA system toxin can influence the virulence of bacteria. However, interestingly, the HigA antitoxin of the *higBA* TA system in *P. aeruginosa* inhibits virulence gene expression [71]. *mvfR* is an important virulence-related regulator in *P. aeruginosa* bacteria, and overexpression of *higA* from its own promoter, located in the upstream toxin *higB,* inhibits the expression of the *mvfR* gene by binding to its promoter region. In antibiotic stress, when Lon protease-mediated degradation of HigA antitoxin increases, there is an increase in the expression of *mvfR* as well as virulence.

The *rhs* locus in the *Salmonella* Typhimurium chromosome is a type II TA system that can modulate host inflammatory responses and the virulence of this organism [73]. There are two copies of the *rhs* TA operon in the *Salmonella* Typhimurium chromosome, termed the main and orphan *Rhs*. *Salmonella* Typhimurium lacking both copies are completely attenuated in pig and cattle infection models [72], and deletion of the orphan toxin alone can reduce *Salmonella* Typhimurium proliferation in mice [74]. Furthermore, it was recently found that Rhs toxins repress *Salmonella* Typhimurium proliferation within host macrophages too, with cells lacking both Rhs toxins proliferating 2-times better within macrophages [75].

### 3.3. Type II TAS Associated with Bacterial Biofilm Formation

Biofilms are complex communities of microorganisms that attach to biotic or abiotic surfaces or to each other and are embedded in a self-produced matrix. The biofilm matrix consists of proteins (e.g., fibrin), polysaccharide (e.g., alginate), as well as eDNA. Most bacteria in the environment live in biofilms, forming part of their survival strategy. For example, *V. cholerae* survives in the water environment in biofilms, regulated by quorum sensing autoinducers [94,95]. Biofilm-residing bacteria can be resilient to both the immune system, antibiotics, and other treatments; therefore, many chronic infections with pathogenic bacteria are associated with biofilm formation [96]. For example, chronic infections caused by *S. aureus, P. aeruginosa* and *M. tuberculosis* bacteria are all associated with biofilms [96,97,98]. Formation of biofilms by pathogenic bacteria is a well-controlled process regulated by environmental cues and quorum sensing associated genes, with multiple steps involved, including initial attachment to the surface, maturation of the biofilm, and detachment of cells and dispersal [99]. TA systems also influence the development of biofilms in several bacterial species (Table 2), although the direct role of TA systems in bacterial biofilm formation has long been debated. It was reported that the type II TA system *mqsRA* was involved in the cross-talk between the *E. coli* quorum sensing autoinducer AI-2 molecule and the production of curli amyloid fibers that play a major role in the formation of biofilms [100,101,102,103]. However, a recent study [104] failed to identify the role of *mqsRA* in any of the functions mentioned earlier.

Other studies confirmed the role of different TA systems in biofilm formation (Table 2). Among them, the very well-studied chromosomal type II TA system *mazEF* has been reported to induce biofilm formation in *E. coli*. It is hypothesised that programmed cell death of the majority of the population occurs via the toxin MazF, allowing the survival of a small subpopulation of cells in the biofilm community by releasing nutrients from the dead cells. It was also reported that the deletion of *mazEF* and *dinJ-yqfQ* from the chromosome decreased biofilm formation in *E. coli* [105,111]. On the other hand, *S. aureus mazEF* inhibits biofilm formation but promotes biofilm antibiotic tolerance, which allows *S. aureus* to transition from an acute infection to a chronic infection that cannot be eradicated with antibiotics [63].

The Enterobacteriaceae plasmid-borne TA system *parDE* is involved in the formation of biofilms in *E. coli* [8], with ectopic expression of the toxin *parE* promotes biofilm formation. Similarly, *higBA* of *P. aeruginosa* reduces the intracellular levels of c-di-GMP by upregulating the expression of c-di-GMP hydrolysis genes, which are associated with the repression of biofilm formation [59,109]. *yqcGF* of *Bacillus subtilis*, as well as *yefM-yoeB* and *relBE* of *Streptococcous pneumoniae*, all promote biofilm production [107,110]. Both the deletion of only *yefM-yoeB*, as well as of *yefM-yoeB* and *relBE* exhibited a significant reduction in their ability for biofilm formation. It was reported that *relBE* family TAS contributes to the complex modulation of the biofilm developmental process in *V. cholerae* [67]. The small chromosome of *V. cholerae* has seven *relBE* TA homologues, and deletion of each of the individual *relBE* loci revealed that *relBE* systems are involved in biofilm formation. *relBE-1* and *relBE-4* deletion mutants produced significantly less biofilm than the wild type. This was corrected after two days, suggesting that biofilm formation may be delayed in these mutants. The *relBE-7* mutant also showed significantly decreased biofilm formation, suggesting that *relBE-7* may be involved in biofilm maturation [67]. *hipAB* of *Shewanella oneidensis* promotes biofilm production presumably by releasing extracellular DNA (eDNA). Interruption of the *hipA* gene by transposon mutagenesis decreases biofilm formation in *Shewanella oneidensis* MR-1 strain [112], and in *E. coli* it was related to the release of eDNA, which is essential as an adhesion molecule for biofilm formation [106].

The well-characterised type II TA systems *hipAB* and *ccdAB* have also been found to contribute to the induction of biofilm formation in probiotic *E. coli* Nissle 1917 [113]. Transcriptional silencing of *ccdAB* and *hipAB* significantly reduced biofilm formation in the Nissle *E. coli* strain. Promoting biofilm formation is very important for probiotic bacteria, as biofilms promote colonisation of the cells onto the gastrointestinal tract mucosal layer, thereby allowing probiotics to develop in situ and carry out functional therapies. It was found that biofilm formation increased the resistance abilities of *Lactobacillus* strains to temperature, gastric pH and mechanical forces [114], and bile-induced biofilm formation during stationary growth allowed *Bifidobacteria* strains to establish strong colonisation in the gastrointestinal tract [115]. Other examples of TA systems involved in biofilm formation include *Rv2871-Rv2872* in *M. tuberculosis* [108] and RelE and VapC toxin homologues in the opportunistic pathogen *Burkholderia cenocepacia* [116].

### 3.4. Role of Type II TA Systems in Bacteriophage Resistance to Bacteria

Toxins of most type II TA systems are RNA endoribonucleases that can cleave mRNAs at specific sites. Bacteria utilise this mRNA cleavage activity against invading bacteriophages to protect them from phage attack. The chromosomal type II TA system RnlA-RnlB of *E. coli* K-12 [117] and plasmid-mediated type II TA system LsoA-LsoB of enterohemorrhagic *E. coli* O157:H7 [118] have anti-phage activity against T4 bacteriophages. The endoribonuclease toxins RnlA and LsoA were upregulated after T4 infection, and the unstable antitoxins RnlB and LsoB disappeared soon after, as T4 infection shuts off *E. coli* gene expression [119]. Therefore, the free toxins RnlA or LsoA degrade most of the T4 mRNAs at the late stage of the infection and inhibits *dmd*-negative T4 phage propagation [117,120,121]. Phylogenetic analysis identified that RnlA domain-containing proteins are also present in other Enterobacteriaceae species, including *Klebsiella pneumoniae*, *Acinetobacter baumannii* and *Pseudomonas aeruginosa* [122]. This suggests that the RnlA-RnlB TA system-mediated anti-phage activity is a common strategy for members of the Enterobacteriaceae family. The chromosomal type II TA system *mazEF* also protects the cell from phage attack, inhibiting P1 phage infection. Studies suggest that deletion of *mazEF* not only enhances the P1 phage production [123] but also significantly increases the propagation of T4 bacteriophages [119]. Another TA system named the *sanaTA* system in *Shewanella* spp. provides resistance against T7 phage lacking the *4.5* gene [124].

### 3.5. Bacteriophage-Borne Type II TA or Antitoxin Helps Phage Propagation in the Host Bacteria

At the same time, bacteriophages have also acquired arms to fight against bacterial TA system-mediated phage defence. To impede the TA-mediated bacterial phage resistance mechanism, bacteriophages have been identified that include antitoxins in their genome as a method of inhibiting host toxins. The well-studied T4 phages evolutionarily obtained the *dmd* antitoxin in their genome that inactivates toxins of both the RnlA/RnlB and LsoA/IsoB type II TA systems of *E. coli* K-12 and O157:H7 [118]. The *dmd* gene is expressed immediately after T4 infection and binds to RnlA or LsoA to inactivate toxin activity, acting as an antitoxin like RnlB or LsoB, resulting in normal propagation of T4 phage [121,125]. This protein Dmd exists in almost all Enterobacteria phages [125] and may provide those bacteriophages with the ability to infect their bacterial host and propagate successfully. Another gene, *ADP-rybosyltransferase* (*Alt*) encoded by the T4 phage, has antitoxin-like activity against MazF, the endoribonuclease toxin of the *mazEF* TA system [119]. The mechanism for the inactivation of the toxin is completely different to that of the Dmd antitoxin. The T4 Alt protein ribosylates MazF toxin immediately after infection of T4 phage, and this modification inactivates MazF by reducing its RNA cleavage activity. Like the T4 bacteriophages, T7 bacteriophages also carry a gene usually annotated as *4.5*, which can inactivate the *sanaTA* system in *Shewanella* spp. [124]. Gp4.5 is an 89-aa glycoprotein with no homology, and *E. coli* K-12 expressing *sanaTA* is resistant to T7 phage lacking the *4.5* gene. The T7 Gp4.5 neutralises *sanaTA*-mediated anti-phage mechanisms by inhibiting the Lon protease activity, thus preventing antitoxin degradation and toxin activation.

The Type II TA system *pfiT-pfiA* found in the Pf4 prophage genome of *P. aeruginosa* not only stabilises the prophage but also regulates the production of active phage virions and phage immunity [126,127]. One study identified that *pfiT-pfiA* inhibits the production of phage particles by inhibiting the phage replication initiation protein [126]. The deletion of the toxin gene *pfiT* greatly increased Pf4 phage production and also activated the expression of phage replication initiation protein gene *PA0727*. However, another study found the opposite function, concluding that the overexpression of the toxin *pfiT* increased the virion production by ~280 folds compared to uninduced strains [127]. It was also suggested that the PfiT toxin triggered the production of new virions and increased their release by lysing bacteria.

### 3.6. Stress Responses Mediated by Type II TA Systems

While the involvement of TA systems in functions discussed above are clear, their role in bacterial responses to various environmental stresses is the subject of much debate. The discovery of TA loci on bacterial chromosomes led to much speculation as to their functions [128]. However, it was found that chromosomal type II TA systems are involved in different kinds of stress management in bacteria, including antibiotic-treated persister formation, tolerance to oxidative, nutritional and heat stresses.

#### 3.6.1. Antibiotic Tolerance and Persister Formation

Chromosomal type II TA systems have been linked to antibiotic tolerance particularly through the formation of persister cells [129,130]. Persister cells are slow-growing, physiologically dormant cells that withstand antibiotic treatment by shutting down the usual cellular processes targeted by antibiotics [130,131]. The link between persister formation and type II TA systems was first established in 1983 when Harris Moyed and Kevin Bertrand isolated a *hipA7* mutant, which bears two substitutions in the HipA protein (G22S and D291S) and exhibits increased persistence up to ~10,000-fold in comparison to the wild-type *E. coli* K-12 strain upon ampicillin treatment [132]. Later, the *hipAB* locus was identified as a TA system [133,134], and it was also demonstrated that the overexpression of the toxin HipA, part of the *hipAB* TA system [132,135,136] led to high rates of persister formation, while deletions of the *hipAB* module decreased persister numbers [135,137]. The ampicillin-selected *hip* mutants also presented increased persistence to quinolone and β-lactam antibiotics [138,139].

After the discovery of the role of *hipAB* in persister formation, type II TA systems with similar toxin-antitoxin properties became favourite candidates for the study of their association with persister development. The *E. coli* chromosome contains more than 36 TA systems; among them, there are 10 endoribonuclease toxin encoding type II TA systems and their role in persister formation has been extensively studied. It was found that individual deletion of them did not affect the persister numbers, but deletion of multiple TA systems up to 10 significantly decreased persister numbers [140]. Although these data were confounded by prophage contamination and experimental conditions [141], studies of independently constructed strains with 5 or 10 TA system deletions from the *E. coli* K-12 chromosome corroborated these findings, concluding that there were no differences in survival of the deletion strains following treatment with several different antibiotics and no link between these TA systems and persistence [142,143].

Given the sequence diversity and specialisation within the TA system family and the known diversity in mode of action [15,33], it is still possible that certain systems can promote persister cell development and other type II TA systems have been implicated. Overexpression of RelE or MazF toxins can strongly promote persister formation in *E. coli*, suggesting a role in conditions when their cognate antitoxins are degraded [137,144]. Deletion of *mqsRA* [145,146] or *yafQ* [147] has been shown to reduce the survival of *E. coli* under antibiotic exposure, suggesting that these TA loci contribute to bacterial persistence. Other examples include the TA systems RelE/RHH-like and BrnTA in *Brucella* spp., with the expression of these genes increasing following exposure to sub-MIC concentrations of gentamicin [148]. Chromosomal *mazEF* in *Staphyloccocus aureus* is also thought to play a role in β-lactam resistance through regulation of other genes, with *mazEF* deletion mutants exhibiting increased susceptibility to penicillin [149]. Activation of TA modules enhancing persister levels in *M. tuberculosis* by host cholesterol molecules has also been reported [150], demonstrating that many factors can influence persister formation.

Like chromosomal TA systems, plasmid-borne type II TA systems can also help to manage the bacterial stresses that induce persisters and increase bacterial survival during infection [84]. Some have also speculated that rather than having separate roles, the functions of plasmid- and chromosome-encoded TA systems actually overlap [151]. Indeed, evidence has also been found for the roles of TA systems in both locations in stress responses, including through other mechanisms. Transient activation of the *E. coli* F-plasmid-encoded CcdB toxin enhances the generation of drug-tolerant persister cells, and this process was found to be dependent on Lon protease and RecA [11]. Another plasmid-borne RelE/ParE superfamily related TA system, *ParDE^I^*, is involved in increased *E. coli* survival rates during treatment with ciprofloxacin, gentamicin and cefotaxime [8]. There is evidence that this is due to activation of the SOS response, as the expression of the ParE^I^ toxin increased the expression of the SOS related genes *recA* and *lexA* [8]. This is in keeping with earlier results showing increased expression of RecA following activation of the toxin CcdB [11]. The F-plasmid-encoded *ccdAB_F_* and IncI and IncF plasmid-encoded *parDE*^I^ have been well-established as a plasmid maintenance system [8,20], and the finding that it plays a role in persistence expands its function as a transmissible persistence factor [8,11]. Type II TA systems found in both chromosomes and plasmids may participate in SOS-induced stress management associated with DNA damaging agents like quinolone antibiotics. The *Caulobacter crescentus* chromosomal *higBA* [152] and recently we have reported that [153] the plasmid-mediated *higBA* type II TA system in Enterobacteriaceae has lexA binding sites (SOS box/lexA box) on their promoter regions. In normal growth conditions, LexA dimers bind to the operator region and repress the expression of *higBA* TA. In the presence of a DNA damaging agent (e.g., ciprofloxacin), RecA stimulates LexA autoproteolysis to derepress LexA-regulated genes and should result in increased expression of *higBA*, which may participate in the management of SOS induced stress response.

#### 3.6.2. Oxidative Stress

Another highly debated topic is the involvement of TA systems in oxidative stress responses. The antitoxin MqsA is linked to the general stress response (GSR) in bacteria [154] and may thus influence cell survival during oxidative stress. The GSR is a reversible state that bacteria can enter in response to a broad range of environmental stresses, including oxidative stress. It is controlled by the inducible sigma factor σ^S^ or σ^38^, encoded by *rpoS*, which is itself regulated by a number of different pathways responding to the different stresses [155]. In normal physiological conditions, MqsA binds to *mqsRA*-like sites in the promoter region of *rpoS*, inhibiting transcription of *rpoS*. During periods of oxidative stress, MqsA is degraded by Lon proteases, thus allowing the expression of *rpoS* to trigger the GSR [154]. However, a recent study found that *mqsRA* deletion strains had similar *rpoS* promoter expression levels to the wild-type strain, as well as similar survival rates following hydrogen peroxide or methyl viologen treatments to induce oxidative stress [114], suggesting that *mqsRA* is not actually involved in the management of oxidative stress. A study of 6 type II TA systems in *K. pneumoniae* (two variations of *relBE, hipAB, vapBC*, *phd-doc* and *mazEF*) found no changes in their expression levels following exposure to oxidative stress, suggesting that these TA systems are not involved [156].

In contrast, it was clearly demonstrated that two type II TA systems *yefM-yoeB* and *relBE* are involved in oxidative stress responses in *S. pneumoniae*. Deletion of the individual or both TA systems significantly reduced the *pneumococcal* survival after exposure to hydrogen peroxide (H_2_O_2_) [110]. Genetic complementation of both TA systems restores *pneumococcal* survival. Protection from H_2_O_2_ killing is very important for *pneumococcal* infection in the human host. When *S. pneumoniae* colonises a new niche, like the lungs, one important hurdle that the bacterium encounters is the presence of free reactive oxygen species (ROS). Thus, hydrogen peroxide (H_2_O_2_) released from the human cells is a key element to be overcome by the invading bacteria [157,158].

#### 3.6.3. Nutritional Stress

The role of TA systems during nutritional stress is comparatively less contentious. Transcription of both *relBE* and *mazEF* has been found to increase during amino acid starvation, leading to inhibition of translation [159,160]. Rather than cell death, the cell enters a state of growth inhibition that allows it to survive starvation which, upon encountering nutrient-rich conditions once more, can be reversed. These results were somewhat contradicted by a study in *E. coli* with 5 TA systems deleted (*mazEF*, *relBEF*, *chpB*, *yefM-yoeB* and *dinJ-yafQ*) [161], in which the authors concluded that the effect of the TA systems was bacteriostatic and not bactericidal and that the absence of these 5 TA systems was no disadvantage to cells in nutrient-limited conditions. It was proposed that this may be due to the effects only occurring following longer starvation exposure times or because the TA systems benefit the cells by reducing translation errors, which would also only become evident over longer time periods [161,162].

#### 3.6.4. Heat Tolerance

The effects of TA systems on heat tolerance in bacteria is relatively less studied compared to other environmental stresses; however, evidence of TA system effects on survival rate during exposure to increased temperatures has been found. *ParDE^I^*, a RelE/ParE superfamily TA system found on IncI and IncF plasmids, increases cell survival at 42 °C, with the induction of both the entire *ParDE^I^* operon as well as only the toxin ParE^I^ having the same effect [8]. The mechanisms of these effects are at present unclear; however, it has been theorised that it is due to similar grown inhibition effects as observed with other environmental stresses.

Another study found single-gene deletions of both the toxin *yafQ* and its cognate antitoxin *dinJ* increases cell survival at 55 °C by approximately 10-fold. The authors attributed this to increased persister cell production and the effects of this TA system on indole production, a signalling molecule that influences the expression of a wide range of genes [163,164]. *yafQ* is an endoribonuclease that degrades transcripts of *tnaA*, which encodes a tryptophanase that converts tryptophan to indole [165]. Indole production was found to decrease heat tolerance; however, somewhat contradictorily, the *ΔyafQ* mutant, which would be expected to have increased TnaA and therefore increased indole production, also had higher heat tolerance than the wild type. This was thought to be due to the concentration of indole in the media, as it has previously been found that similar concentrations of indole increased persister frequency in *ΔtnaA* mutants [164,166].

#### 3.6.5. Other Stresses

Evidence had also been found of the role of TA systems in other environmental stresses. Expression of the TA systems *relE/RHH-like*, *fic/phd* and *brnTA* increased following exposure of *Brucella* spp. strains to acid shock conditions (pH 5.5), suggesting a possible role for these TA systems in acidic conditions [148]. *PemIK* has also been shown to influence biofilm formation in the *Klebsiella pneumoniae* strain ST846-OXA48CA, as it was found on a plasmid within this strain that also shows tolerance to chlorohexidine treatment [167].

## 4. Applications of Type II TA Systems in Biotechnology and Medicine

With their diverse biological functions in bacteria, TA systems have significant value in biotechnology, molecular biology and in antibacterial drug design. The toxic properties of TA toxin proteins have been exploited to design tools for molecular cloning for positive selection vectors. One example is a positive selection vector containing a toxin gene, typically *ccdB* from the F-plasmid, that is inactivated upon insertion of foreign DNA [56,168], allowing only insert containing clones to grow. Another example is the StabyCloning^TM^ system using the *ccdAB* system, where the vector contains a truncated version of the *ccdA* antitoxin. Attachment of a 14 bp sequence to the 5’ end of the DNA fragment to be cloned restores the active antitoxin, which neutralises the toxin. Thus, only cells containing a vector with an insert in the desired orientation can form colonies [168]. Plasmid instability is another major problem in producing a single protein from the expression vector during the fermentation process. Antibiotic selection pressure must be maintained during the whole fermentation process to maintain the plasmid and get maximum yield, which is costly and create a risk of contaminating the protein product with antibiotics and requires special waste treatment to avoid the release of antibiotics into the environment being contaminated. This problem can be solved easily by using TA system to stabilise plasmids in the antibiotic-free growth environment [169,170,171].

TA systems can be used to design antibacterial drugs to fight against antibiotic-resistant bacteria, as overexpression of toxin can kill or inhibit the growth of bacteria. Many toxins of type II TA systems perform this bacterial killing/growth inhibition by inhibiting bacterial translation, actions similar to some antibiotics. Therefore, artificial activation of toxin or inactivation/degradation of the antitoxin could be a potential approach to achieve this goal. Antibiotic resistance or virulence plasmids can be cured of bacteria by using plasmid incompatibility, but plasmid-free cells can be killed off by stable toxins from plasmid-mediated TA systems. To circumvent this problem, curing plasmids have been constructed to provide excess antitoxin in trans by engineering TA modules (deletion of toxin gene) with plasmid incompatibility to cure target antibiotic resistance plasmids in vitro and in vivo successfully [172,173]. This opens a new area for selective neutralisation of TA modules to cure antibiotic resistance and virulence plasmids without killing bacterial populations.

## 5. Limitations in TA Research

Despite increasing interest in TA system research, there are several limitations in the TA research that we should anticipate. **(i) TA nomenclature**: the naming system is a significant issue in the TA research area. Currently, there is no unique and established system for the nomenclature for the newly identified TA system. A proper naming system like ISFinder should provide the unique name by judging their sequence identity, source of origin (e.g., bacterial or archaeal species) and localisation (whether it is located on plasmids, chromosomes or other mobile elements). Otherwise, the current practice of giving the same name to TA systems with a different mechanism of action or biological functions or vice versa will continue. One good example is the RelB and ParE toxins. Both are members of the RelE superfamily with high amino acid sequence identity, but their mechanism of action is different, where RelE is an endoribonuclease and ParE is a gyrase inhibitor [8,174,175]. **(ii) standard protocol for studying TAS**: studies on TA system effects on bacterial growth, survival and different stress conditions should be carried out with a standard and common protocol using well-characterised bacterial strains. Changing experimental conditions, toxin induction time, the concentration of inducer used, and the bacterial strains may influence the results. Other confounding factors like bacteriophage contamination also can affect the experimental results [141]. **(iii) study with whole TA module or deletion mutants instead of ectopic expression of toxin only**: in many published works, the effects of TA systems on bacterial growth and physiology were measured after ectopic expression of toxin gene of TA system only. Artificial expression of the toxin gene can inhibit bacterial growth in most cases. Therefore, measuring physiological changes on already stressed bacteria may not provide the actual results. Consequently, it is ideal for studying TA system functions from the presence or absence of whole TA systems as well as ectopic expression of the toxin gene.

## 6. Concluding Remarks

Bacterial toxin-antitoxin systems initially thought of as mysterious selfish genetic elements are now increasingly acknowledged as important genetic modules that have a significant impact on the regulation of bacterial growth, physiology, virulence and many other functions. Interest in TA research has significantly increased in the past decade, leading to discoveries of new TA systems with completely novel mechanisms of action and roles in bacterial physiology. TA systems on mobile genetic elements not only contribute to their maintenance but could also perform similar functions to that of their chromosomal counterparts [8,11]. The mobility of TA systems associated with MGEs means that TA functions are easily transferred to other bacteria, providing survival advantages in adverse environments, including antibiotic resistance. Despite the controversy on some TA encoded functions, to date, the real biological functions of many of the type II TA systems have been elucidated. However, the *bona fide* functions of a large number of type II TA systems still remains elusive. More research is needed to understand the real functions of both characterised and uncharacterised type II TA systems.

## Figures and Tables

**Table 1:** Type II TA systems involved in bacterial virulence and pathogenesis.

Type II TA	Bacterial Species	Localisation	Function	Reference
*mvpAT/vapBC*	*Shigella species*	plasmid	maintains virulence plasmid at temperature of human intestine	[50,51]
*gmvAT*	*Shigella species*	plasmid	maintains virulence plasmid at 21 °C	[50]
*vapBC_ST_*	*Salmonella species*	plasmid	maintains pSLT virulence plasmid and increases *Salmonella* survival inside host cell	[9,52,53]
*ccdAB_ST_*	*Salmonella species*	plasmid	maintains pSLT virulence plasmid	[52]
*pumAB*	*Pseudomonas aeruginosa*	plasmid	facilicates mouse organ invasion and increases *C. elegans* and *mouse* mortality rate	[54,55]
*pemIk_Sa_*	*Staphylococcus aureus*	plasmid	regulation of virulence gene expression	[56]
*ε/ζ*	*Streptococcus pneumoniae*	chromosome	virulence in mice	[57,58]
*higBA*	*P. aeruginosa*	chromosome	reduces production of virulence factors	[59]
*vapBC3, vapBC4*	*M. tuberculosis*	chromosome	increases pathogenesis in animal model	[60]
*vapBC11*	*M. tuberculosis*	chromosome	essential for infection in guinea pigs	[61]
*mazEF3, mazEF6, mazEF9*	*M. tuberculosis*	chromosome	increases survival in macrophages and increases colonisation in spleen and lung of guinea pigs	[62]
*mazEF*	*S. aureus*	chromosome	helps transitioning from acute to chronic infection	[63]
*vapBC-1,* *vapXD*	*Haemophilus influenzae*	chromosome	increases survival inside epithelial cells and in the ear of infected chinchillas	[64]
*toxAvapA*	*H. influenzae*	chromosome	role in chinchilla middle ear infection	[65]
*ybaJ-hha, yefM-yoeB*	*E. coli*	chromosome	contributes to the colonisation in the bladder	[66]
*pasTI*	*E. coli*	chromosome	contributes to the colonisation in the kidneys	[66]
*relBE4,* *relBE7*	*V. cholerae*	chromosome II	improves intestinal colonisation in mice	[67]
*fitBA*	*Neisseria gonorrhoeae*	chromosome	intracellular growth regulator	[68]
*pezAT*	*S. pneumoniae*	chromosome	virulence in mice	[58,69]
*higBA*	*P. aeruginosa*	chromosome	reduces virulence factors production	[59]
*SehAB*	*Salmonella enterica*	chromosome	reduces virulence in mice	[49]
*savRS*	*S. aureus*	chromosome	negatively regulates the virulence gene expression and pathogenicity	[70]
*higBA*	*P. aeruginosa*	chromosome	inhibits virulence gene expression	[71]
*Rhs locus*	*Salmonella* Typhimurium	chromosome	represses proliferation within host macrophages	[72,73,74,75]

**Table 2:** Type II TA systems involved in bacterial biofilm formation.

Type II TA	Bacterial Species	Localisation	Function	Reference
*parDE*	*E. coli*	plasmid	promotes biofilm formation	[8]
*mqsRA*	*E. coli*	chromosome	biofilm production/controversial	[104]
*mazEF and dinJ-yqfQ*	*E. coli*	chromosome	promotes biofilm production	[105]
*hipAB*	*Shewanella oneidensis and E. coli.*	chromosome	promotes biofilm production	[106]
*yqcGF*	*Bacillus subtilis*	chromosome	promotes biofilm production	[107]
*Rv2871-Rv2872*	*M. tuberculosis*	chromosome	enhances biofilm development	[108]
*higBA*	*P. aeruginosa*	chromosome	repression of biofilm production	[109]
*yefM-yoeB, RelBE*	*S. pneumoniae*	chromosome	promotes biofilm formation	[110]
*relBE and variants*	*V. cholerae*	chromosome	promotes biofilm formation and biofilm maturation	[67]
*higBA*	*P. aeruginosa*	chromosome	reduces biofilm formation	[59]
*mazEF*	*S. aureus*	chromosome	promotes biofilm formation	[63]

## Data Availability

Data is contained within the article.
